# Circadian Rhythm and Stress Response in Droppings of* Serinus canaria*

**DOI:** 10.1155/2016/3086353

**Published:** 2016-12-25

**Authors:** Maura Turriani, Nicola Bernabò, Barbara Barboni, Gianluca Todisco, Luigi Montini, Paolo Berardinelli

**Affiliations:** ^1^Unit of Basic and Applied Bioscience, Faculty of Veterinary Medicine, University of Teramo, Via Renato Balzarini 1, 64100 Teramo, Italy; ^2^Via per Mosciano, No. 96, Giulianova, 64021 Teramo, Italy; ^3^Via Villafranca No. 11, 72100 Brindisi, Italy

## Abstract

*Serinus canaria* is a widespread domestic ornamental songbird, whose limited knowledge of biology make compelling studies aimed to monitor stress. Here, a commercial enzyme immunoassay was adopted to measure immunoreactive corticosterone (CORT) in single* Serinus canaria* dropping sample, to monitor the daily fecal excretion of CORT in birds bred singly or in-group and to detect the effect promoted by aviary or small transport cage restraint. A robust daily rhythm of CORT was recorded in animals held on short-day light cycle, independent of bred conditions (single or group), which persisted when space availability was modified in single bred animal (transfer in aviary and transport cages). By contrast, a significant change in CORT excretion was recorded when group bred animals are restrained in a smaller cage. The daily rhythm in CORT excretion in response to manipulation showed the greatest response at the beginning of the light period, followed by the absence of the peak usually recorded at the end of the dark phase. These data indicated that EIA could be used as a reliable noninvasive approach to monitor the stress induced by restraint conditions in* Serinus canaria*.

## 1. Introduction

The* Serinus canaria* is a small domestic songbird native of Canaria Islands that is usually maintained in captivity for ornamental purposes. Despite its spread and the increasing evidences demonstrating that stress is related to several environmental and social conditions as well the human-induced disturbances, scarce information on the physiology of this bird is still available. Stress plays an important role in the maintenance of body homeostasis, conditioning health, and breeding outcomes. It is known that CORT are front-line hormones to overcome stressful situations. During short-term stress, they improve fitness by energy mobilization [1, 2] and regulate behavior and suppress metabolic processes nonessential for survival. However, severe or chronic stress may decrease individual fitness by immunosuppression and atrophy of tissues [3, 4] and the decreases of reproductive performance [5, 6] and there are also indications of stereotypies [7].

In the past, CORT have typically been quantified in blood [8–14] while, more recently, noninvasive methods are being developed and applied to several avian species, since they offer several advantages [15–25]. In particular, fecal steroid hormones assays are now being used in a variety of disciplines (e.g., animal science, behavioral ecology, conservation biology, ornithology, and primatology) to examine the reproductive and adrenocortical status of a variety of taxa [12, 16, 21, 22, 26–39]. These noninvasive methods allow easy collection of samples without disturbing the animals. Samples can be collected at regular intervals through time, thus providing an accurate assessment of stress without the bias of capture-induced increases in CORT [22, 31, 40, 41]. Differently from blood CORT, steroids in feces are cumulative thus provide a measure of circulating CORT over time [23, 31, 42, 43].

Although use of noninvasive techniques has increased for many species, several confounding factors must be considered before using them: the specie-specific modality of CORT excretion, the daily [44, 45] and seasonal patterns [11, 46, 47], the reproductive status [11, 48], the sex [21], the body condition [49, 50], and the photoperiod [51, 52]. In addition, steroids such as CORT are metabolized in the liver and excreted into the gut through bile [20, 22, 24] and are present in the feces either as metabolites [51, 52] or native hormones.

Fecal CORT assays have been validated in several species [16, 22, 53] but only fewer studies have focused on parrots [54, 55] or small (<200 g body mass) birds [15, 56]. In this context, no information regarding normal values of excreted CORT in* Serinus canaria* is now available despite the widespread diffusion of this breeding.

The possibility to enlarge the use of fecal CORT measurement for detecting the wellbeing in birds is limited by the validation of radioimmunoassay (RIA), in spotted owls [57] and in stonechats [58].

To detect minute quantities of feces commercial EIA kits can be adopted, due to their advantages in terms of costs, ease of use, and availability.

A negative relationship between fecal sample mass and fecal CORT concentration has been found previously [23, 40], leading to the suggestion of only analyzing larger fecal samples (>20 mg dried mass). This may potentially preclude the analysis of individual samples from many species of smaller birds, which typically produce a fecal sample smaller than 20 mg [16]. This problem could be overcome by combining multiple fecal samples or collecting them over a larger windows of time [31, 40, 59, 60]. Starting from these premises, the present research has been designed in order to test if a commercial EIA, previously used to assay CORT in zebra finches [61], can be adopted to detect fecal CORT at individual level.

Once assay has been adapted to record CORT in* Serinus canaria* feces and tested for its analytical performances (parallelism, intra- and interassay variation, and sensitivity) and biological reality (oral corticosterone challenge), the EIA was used to record the basal daily secretion of CORT in adult females (1-year olds) bred into conventional cages, during a short-light period, at stable 20°C of environmental temperature and feed ad libitum.

First, social effect has been evaluated by comparing fecal CORT excretion in individually* versus* group bred birds. Then, the effect of spatial availability has been determined by transfer bird bred individually or in group into smaller (conventional transport cages) or lager cages (aviary).

## 2. Materials and Methods

All research was approved by the ethical committee of the University of Teramo even if the experimental plan reproduces conventional animal management.

### 2.1. Animal Study

Adult, female bred* Serinus canaria* were housed under control conditions in cages of 55 × 60 × 90 cm individually or in group (4 birds) to detect the basal levels of excreted CORT collected during 24 h. They were given water and a commercial seed diet ad libitum and housed on short day photoperiod (a 10 hours of light/14 dark cycle). The animals were maintained under stable temperature of 20°C.

For the biological validation, 10* Serinus canaria* bred individually were randomly divided in two experimental groups: Ctr and oral corticosterone challenged birds according to previously validated protocols [58, 62, 63].

To this aim* Serinus canaria* were fed with exogenous corticosterone suspended in peanut oil (1.0 mg/mL). Corticosterone solution was administered orally in a single morning dose of 0.1 mL while Ctr were feed with oil alone. Dropping were sampled four times after corticosterone administration performed at 7.00 am (time 0: prior to administration and times 3, 6, 9, and 24 indicated the hours after administration).

The experimental trials were performed singly (*n* = 5) or in group (*n* = 5 for 4 birds each) held birds; they were caught by hand and placed individually or in group, respectively, into smaller transport cages (15 × 60 × 30 cm) or in aviary (120 × 100 × 100 cm) and then transferred again into control cages the day after.

During the experimental periods all droppings excreted by animals were collected at 3 h intervals (from 10.00 am of the first day until 10.00 am of the following day). In order to facilitate dropping collection, the bottom of the cages was lined with plasticized sheet. Whole excreted samples deprived only of feathers/food contaminants were immediately deposited in cryotubes, identified, and stored at −20°C.

Then the birds were transferred into their original bred condition.

The dropping were stored frozen at −20°C until the last collection when the extraction procedure was performed.

### 2.2. Preparation of Droppings

Before assay, droppings were thawed at room temperature, placed in glass tubes, and weighed wet. Samples were dried in a forced-air oven at 90°C for 1 h to destroy bacterial enzymes [24, 25]. The samples were ground, homogenized, and weighed.

Glucocorticoids were extracted by first normalizing in each experiment the weight of dropping to extract on the minimum weight obtained amongst samples. In this way, within each experiment, a similar amount of dropping/samples were extracted with 75% methanol and 25% double distilled water normalizing the volume on the basis of sample weight (max 2 mL) followed by vigorous vortexing for 5 min, centrifugation (2.300 ×g, 15 min, 4°C), and freezing (−85°C for 15 min).

Centrifugation and freezing that resulted in the solidification of the samples at the bottom of tube are required to decant the supernatant methanol in another grass tube (12 × 75 mm). The supernatant was THEN evaporated under a stream of air at 90°C. Finally, we added 0.01 M Phosphate Buffered Saline with 0.1% gelatin to each tube and vortexed them vigorously for 5 min, and then assay was carried out.

### 2.3. Immunoassay Procedures and Validation

In the present study, the Enzo Enzyme Immunoassay kit (Enzo Life Science; Cat n° ADI900-097) was used. The first step was a standard analytical validation of the commercial EIA on CORT dropping extracts, collected from individual bird realized including assessment of parallelism, intra-assay and interassay precision, and assay sensitivity. To this aim, CORT dropping extracts obtained from samples containing high levels of CORT were used.

Samples were initially spiked with approximately 1 pg of tritiated corticosterone prior to steroid extraction and ~84,6% of recovery was obtained (max coefficient of variation 16,3%) according to results described by [61] by adopting the same commercial EIA on avian blood samples.

For parallelism assessment, curves of percent binding of EIA standard corticosterone (%*B*/*B*_0_) were analyzed* versus *serially diluted high dropping extract samples log-transformed doses 1 : 2 (dilution 1), 1 : 10 (dilution 2), 1 : 100 (dilution 3), and 1 : 200 (dilution 4).

Lastly, a biological test was performed by dosing fecal CORT dropping samples collected from oral corticosterone challenged animals.

The EIA analysis was performed according to manufacturing instructions. The antibody specificity was not internally determined. However, the manufacturers declare for corticosterone EIA antibody 100% cross reactivity, 28% for desoxycorticosterone, 0.3% for tetrahydrocorticosterone, and 0.2% for aldosterone.

### 2.4. Statistical Analysis

Each analytical sample (standard curve points and experimental points) was analyzed in triplicate while each experimental data was the mean of at least five different birds or group of animals. Reported hormone values are based on the average of at least closest two of the triplicate values obtained within each assay, except in cases where these had greater than 10% variation. In such instances, remaining samples was reanalyzed in an additional assay. All the calculations have been performed considering the data as not normally distributed (D'Agostino and Pearson omnibus test), by using not parametrical tests: Mann–Whitney *U* test for two samples or Kruskal-Wallis test for more samples (Past 3). The data are represented as median ± 25°–75° percentile.

## 3. Results

### 3.1. EIA Validation

**Figure 1:**
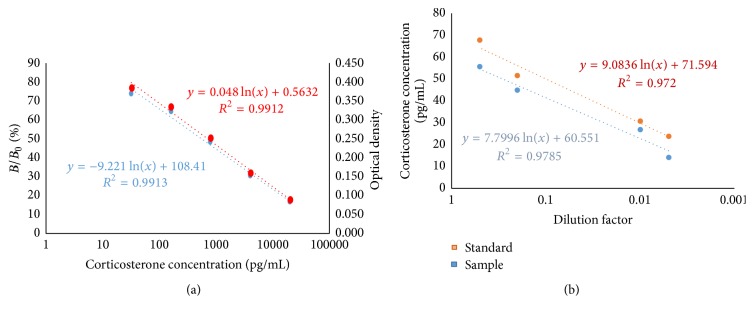
(a) Standard curves. (b) Diagram showing the curves slope at different dilutions (dilution 1 = 1 : 2, dilution 2 = 1 : 10, dilution 3 = 1/100, and dilution 4 = 1/200). *X*-axis in logarithmic scale.

The quality control of assay was obtained by evaluating the standard curves (%*B*/*B*_0_ versus CORT concentration and CORT concentration versus Optical Density) (see Figure 1(a)) in which the quality of fit is always >0.99, whereas the ability of EIA to actually measure the CORT concentrations was assessed by the parallelism assay (see Figure 1(b)). Based on the results of serial dilutions, fecal extracts were usually diluted to 1 : 10 prior to assay.

The EIA sensitivity was ~20 pg/mL. The intra-assay and the interassay coefficients of variation were 10.4% and 16.9%, respectively, similar to those declared by manufacturing guidelines. The reality of EIA protocol applied of individual dropping CORT collected during the interval time of 3 hours is considered for the following experiments since it allowed detection of ~81.2% of the collected samples instead of the 47.4% of samples collected at 1 hour of interval.

### 3.2. Fecal Excretion

Quantities of dropping varied amongst birds (from 0.046 g to 0.121 g/3 h) and they did not change during the days (early morning 0.064 ± 0.021 g/3 h; afternoon 0.096 ± 0.049 gr/3 h, *p* > 0.05: data not shown) in single animals bred under short day photoperiod.

### 3.3. Daily Excretion of CORT

**Figure 2:**
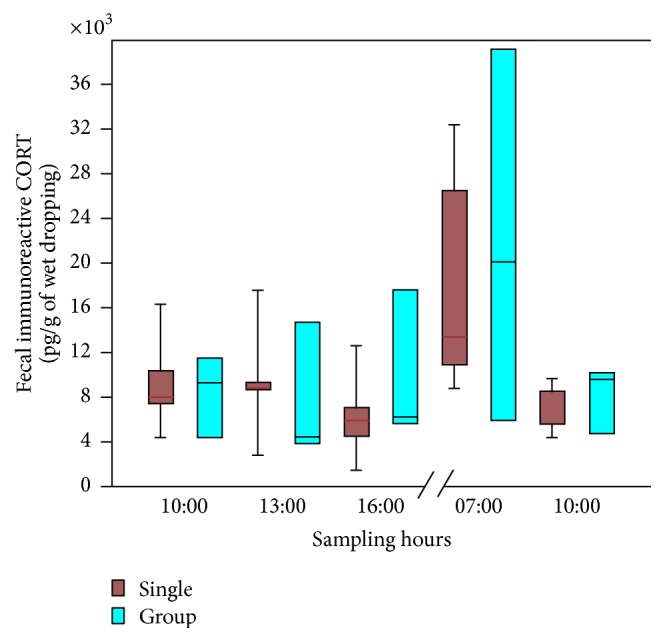
Diagram showing the daily rhythm in CORT excretion either in single (blue) or in grouped (red) bred birds. The sampling was carried out at different times, as reported on *x*-axis. For statistical analysis, refer to the result section of the manuscript.

The four time points (10.00, 13.00, 16.00, and 7.00) analyzed through repeated measures documented a daily rhythm in CORT excretion, either in individual or in group bred birds (Figure 2). On short day photoperiod, basal low levels of CORT were recorded through the light (active) period to increase significantly in dropping samples collected immediately after light came on. The pattern and the levels of CORT excretion over the 24 h interval did not differ between single and in group bred animals (Figure 2).

### 3.4. Oral Corticosterone Challenge Effect on Daily CORT Excretion

**Figure 3:**
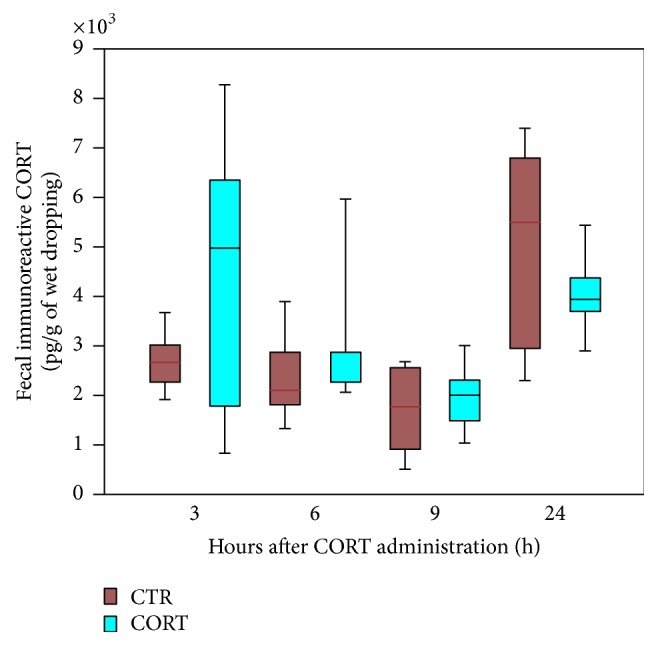
Diagram showing the daily rhythm in CORT excretion in control animals (CTR) or in birds feed with one dose of corticosterone suspended in 0.1 mL of peanut oil (1 mg/mL) (FEED). 3 h, 6 h, 9 h, and 24 h are referred to as the hours after the CORT administration. For statistical analysis, refer to the result section of the manuscript.

The profile of fecal CORT did not change in birds of control (5 animals) feed with peanut oil alone (CTR in Figure 3). Differently, the birds (*n* = 5) feed early in the morning with one dose of corticosterone suspended in 0.1 mL of peanut oil (1 mg/mL) displayed significantly higher level of CORT 3 and 6 hours later (*p* < 0.01 and *p* < 0.05, respectively, Figure 3). The CORT excretion, then, acquired a pattern and levels similar to those recorded under CTR conditions.

### 3.5. Effect of Different Restraint Conditions on CORT Excretion

The fecal CORT concentrations remained unchanged when* Serinus canaria* bred individually or in group were moved into aviary. Birds transfer in a larger cage never modified the daily pattern and the levels of excreted CORT (data shown).

**Figure 4:**
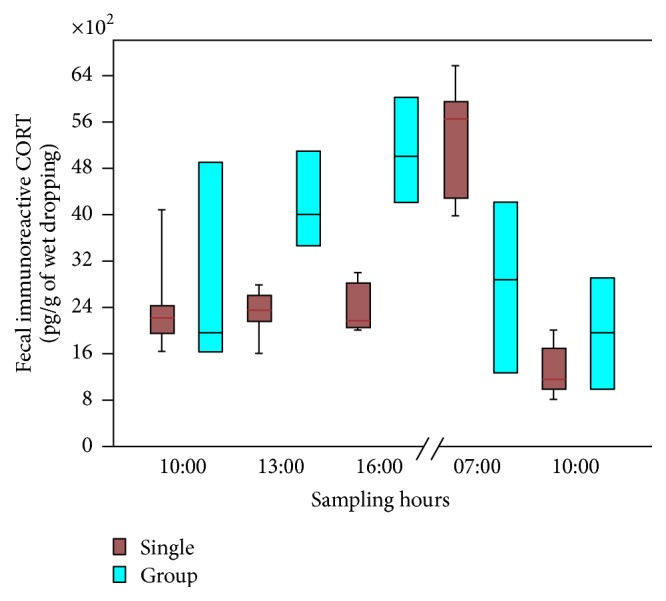
Diagram showing the daily rhythm in CORT excretion measured after bird transfer in smaller cages in single (blue) or in grouped animals (red). The sampling was carried out at different times, as reported on *x*-axis. For statistical analysis, refer to the result section of the manuscript.

On the contrary, the daily dropping levels of CORT measured after bird transfer in smaller cages, as summarized in Figure 4, were conditioned to preliminary bred conditions.

The levels as well the daily circadian excreted CORT remained unchanged in* Serinus canaria* bred individually and moved into small transport cages (Figure 4). Differently, the pattern of CORT excretion was modified when* Serinus canaria* had bred preliminary in group. In this experimental condition, higher levels of dropping CORT levels were recorded at 13.00 and 16.00 (*p* < 0.01* versus* single bred animals). In addition, the early morning peaks usually recorded in samples collected immediately after light came on (7.00: *p* < 0.01* versus* single bred birds, Figure 4) were not observed.

The birds acquired again the daily CORT rhythm the day after they were transferred in group in normal cages (data not shown).

## 4. Discussion

### 4.1. EIA Validation

The present data demonstrated, for the first time, the validity of a commercial double antibody EIA kit for detecting excreted CORT in* Serinus canaria*, probably the most widespread songbird domesticated avian species.

The specificity of EIA was confirmed through the parallelism experiments and by detecting increased fecal levels of CORT in birds orally treated with corticosterone. Unfortunately, the small size of* Serinus canaria* did not allow us to design, in parallel, experiments aimed to pharmacologically induce circulatory changes of CORT, as proposed by Young and Hallford [58].

Analogously, the daily rhythm recorded in single and group bred birds has represented, indirectly, a further approach to validate the use of this commercial kit for monitoring stressful conditions on small amount of dropping collected from single bred* Serinus canaria*.

In this context, the several technical (the absence of radioactivity, minimal equipment requirement, etc.) and biological advantages of a commercial EIA cannot be underestimated when the assay can be adopted, in particular, for practical aims. The high impact of translating stress detection to clinical and breeding practices of widespread species like* Serinus canaria *could justify a qualitative use of the assay (for example, CORT values over basal daily levels) thus underestimating limit imposed by a precise quantitative dosage.

### 4.2. Daily Rhythm

EIA analysis enabled us to describe, for the first time, the CORT daily rhythm of* Serinus canaria,* evaluated on single dropping sample collected from animals bred under short day photoperiod and maintained under controlled bred conditions.

The* Serinus canaria* showed, indeed, a 24-hour cyclic CORT excretion. The repetitiveness of CORT data that constitutes itself as a strong validation of EIA assay was not affected by the bred conditions considered (single* versus* group).

Under short days (10L/14D), basal levels remained low throughout the light period (one sample each 3 h from 10.00 am to 16.00) to peak in before lights came on (7.00). Considering the delay in the feces, it may be presumed that also in* Serinus canaria *the CORT peak in the plasma occurred during or at the end of the dark hours.

The CORT levels during a 3 h interval before light double, even if the birds did not eat during this inactive period. This entails that the increase in CORT rate cannot be attributed to differential food intake while lights are off but to a preactive peak of CORT that was similarly observed in other avian taxa [44, 51, 52, 64–67]. The preactive peak may be necessary to prepare an appropriate physiological state to meet energetic demands as active period begins. In mammals CORT daily rhythm is thought to regulate overall metabolism [9, 68] and, together with insulin, regulates energy acquisition, deposition, and mobilization [69, 70]. A similar assumption has been translated to some birds (e.g., pigeons and chickens) that displayed CORT rhythms similar to that of mammals [8, 10, 71]. Relatively few studies, instead, have evaluated CORT rhythm in passerine such as white-throated sparrow,* Zonotrichia albicollis* [66], Gambel's white-crowned sparrow* (Zonotrichia leucophrys gambelii)* [44], the European stonechat (fecal CORT)* (Saxicola torquata rubicola)* [30], house sparrow* (Passer domesticus)* [45], the starling,* Sturnus vulgaris *[45], the great tit,* Parus major* [51, 52], and more recently in zebra finches [72].

On the basis of fecal CORT diel rhythm, two interesting hypothesizes may be proposed. Firstly, since under two different bred conditions CORT reached similar daily levels in dropping sample before the active period, this finding seems to suggest a potential biological relevance of the peak during the preactive period driving behavioral and physiological processes. Second, like other passerine species, the excreted CORT levels in* Serinus canaria* are very low during the active period, as a consequence of a sharp drop in CORT metabolites occurring immediately after the first hour of the day. This peculiarity has suggested avian-specific mechanisms of regulation. This hypothesis was firstly advanced from Breuner et al. [44] that recorded a rapid decrease in CORT plasma levels during most of active day in Gambel's white-crowed sparrow (differently from mammals [44]). This could not be ascribable to high mass specific metabolic rate since also in smaller mammals, like mice or rat, the blood CORT basal levels are 5–10 times higher than in sparrow [69, 73, 74]. In addition, low active CORT blood levels in avian cannot be due to a greater intestinal clearance since, as documented in the present paper, low and stable fecal CORT levels were similarly recorded at least in the* Serinus canaria*. Even if further experiments investigating the changes in CORT secretion and clearances are required to establish the cause of this rapid decline, a passerine specificity could be hypothesized.

### 4.3. Response to Modified Restraint Condition

As regards the relationship between stress-induced levels and rhythm, it seems that, in most of avian species, the intensity of the CORT response to stressors is dependent on the day at which the stimulus is presented. The stress response has a daily rhythm, which approximately mimics the pattern of the basal fluctuation, at least on a winter photoperiod [44, 45, 51, 52]. This was, partly, confirmed in the* Serinus canaria. *The hypothalamic-pituitary-adrenal (HPA) axis appeared to be sensitive to specific stressor just after light came on, but the daily basal rhythm was slightly impaired under persistent stressor conditions. Moreover, the perception of a stimulus as stressor seems to be strictly conditioned by the preliminary bred condition. Indeed, whereas single bred animal did not modify the CORT basal levels, when they are transferred in cages of different size (aviary and transport cages). By contrast, the transfer in transport cages applied to birds bred in group became able to increase CORT secretion in few hours early in the morning.

The explanation of this different behavior in steroids secretion cannot be easily interpreted based exclusively on fecal CORT dosages. Firstly, since several evidences demonstrated that CORT secretion plays a key role in the consolidation of memories of a stressful events, the subject can respond more effectively when the events reoccur in the future [75, 76]. If this is the case, the lower sensitivity to transport restraint, observed in single bred animal could be interpreted as their more practice to tolerate stressor conditions in comparison to group bred ones that could have experienced greater stressor live conditions (ex. hierarchy within birds). Based on this hypothesis, it is possible to explain, for example, the different levels of CORT recorded in the Bengalese finch (*Lonchura striata* var.* domestica*), a domesticated strain of the white-backed munia (Lonchura striata). The domesticated songbirds have reduced CORT levels because of reduced levels of environmental stresses (compared to wild related species) and reductions in the role of CORT, which is necessary for survival in the wild.

In addition, another mechanism regulating CORT individual can be considered. Indeed, it has recently been demonstrated in mice that the effects of stress on free CORT are stressor specific, with respect both to the magnitude and to the duration of the response and that they are mediated by circulatory corticosteroid binding globulins (CBG). CBG seems to be released from the liver at high levels only in response to stressors of moderate to strong intensity. Thus, the increase in circulating CBG levels after stress restrains the rise in free CORT concentrations in the face of mounting total hormone levels in the circulation. This highly dynamic mechanism involved in the regulation of glucocorticoid hormone physiology complicates the interpretation of CORT fecal secretion levels after acute stress.

However, even if it is quite difficult to interpret the mechanism involved in these different CORT responses, the combination of the group bred and transport restraint condition was able to evoke a significant increase in CORT over basal levels that, however, determine a transitory reduction of the daily rhythm. The day after, indeed, the preactive morning CORT peak was significantly inhibited. Interesting, the modification of space in not per se a condition sufficient to evoke stress in* Serinus canaria* since restraint in larger cages that did not modify CORT secretion. Exclusively the limitation of space may determine a CORT response when, however, it was applied to group bred birds.

The rapid reduction of fecal CORT levels can offer a protective response, able to limit the long term adverse effects that chronic elevations can have on the brain, such as hippocampal atrophy, increased feeding rate, and immune function [4].

Instead, the detrimental effects of prolonged low concentration of systemic CORT levels have not yet studied in avian species even if exhaustion of glucocorticoid response may represent a very critical situation for animals homeostasis that, however, should take place under pathological conditions (endocrine syndrome) or after more prolonged stressor exposure.

## 5. Conclusion

The availability of commercial EIA for the noninvasive monitoring of stress in captive widespread bird species like* Serinus canaria* has a large clinical and breeding management impact. The identification of an endogenous CORT variations over the 24 h is essential to interpret the stressor effects and to approach future focused experiments. The description of the baseline circulating CORT fluctuates on a diel basis would, indeed, allow researchers to remove that source of variation by planning experiments at appropriate time of day.

The experimental results suggested, in addition, that domesticated* Serinus canaria *bred individually does not experience stressor modified restraint conditions (aviary or transport cage transfer). On the contrary, higher attention must be addressed to the management of* Serinus canaria* bred in group in order to prevent stress response determined by the reduction of cage space.
